# A Hybrid Decision-Making Approach for the Service and Financial-Based Measurement of Universal Health Coverage for the E7 Economies

**DOI:** 10.3390/ijerph16183295

**Published:** 2019-09-07

**Authors:** Xiaofeng Shi, Jianying Li, Fei Wang, Hasan Dinçer, Serhat Yüksel

**Affiliations:** 1 School of Physical Education, Shanxi University, Taiyuan 030006, China; 2 Sports Science Institute, Shanxi University, Taiyuan 030006, China (J.L.) (F.W.); 3 School of Business, İstanbul Medipol University, Istanbul, 34810, Turkey

**Keywords:** universal health coverage, E7 countries, fuzzy DEMATEL, MOORA

## Abstract

The aim of this study is to measure universal health coverage in Emerging 7 (E7) economies. Within this framework, five different dimensions and 14 different criteria are selected by considering the explanations of World Health Organization and United Nations regarding universal health coverage. While weighting the dimensions and criteria, the Decision-making Trial and Evaluation Laboratory (DEMATEL) is considered with the triangular fuzzy numbers. Additionally, Multi-Objective Optimization on the basis of Ratio Analysis (MOORA) approach is used to rank E7 economies regarding Universal Health Coverage (UHC) performance. The novelty of this study is that both service and financial based factors are taken into consideration at the same time. Additionally, fuzzy DEMATEL and MOORA methodologies are firstly used in this study with respect to the evaluation of universal health coverage. The findings show that catastrophic out of pocket health spending, pushed below an international poverty line and annual growth rate of real Gross Domestic Product (GDP) per capita are the most significant criteria for universal health coverage performance. Moreover, it is also concluded that Russia is the country that has the highest universal health coverage performance whereas China, India and Brazil are in the last ranks. It can be understood that macroeconomic conditions play a very significant role on the performance of universal health coverage. Hence, economic conditions should be improved in these countries to have better universal health coverage performance. Furthermore, it is necessary to establish programs that provide exemptions or lower out-of-pocket expenditures which will not prevent the use of health services. This situation can protect people against the financial risks related to health expenditures. In addition to them, it is also obvious that high population has also negative influence on the countries such as, China and India. It indicates that it would be appropriate for these countries to make population planning for this purpose.

## 1. Introduction

Universal health coverage refers to the healthcare services that are provided to all citizens. In other words, it means that all people in the country can access the different types of healthcare services which are necessary for their lives [1]. In this definition, it can be understood that universal health coverage includes the citizens who do not have financial power to pay for healthcare services. Thus, almost all countries in the world try to take some action to achieve an effective universal health coverage system [2]. With the help of this issue, it can be possible to increase the quality of the life of the citizens.

It is obvious that the determinants of the universal health coverage should be identified to achieve this objective. Within this framework, it is believed that antenatal and delivery care and full child immunization provide information about the performance of universal health coverage [3]. In addition to these factors, the number of hospitals and doctors and the access to essential medicines are accepted as the important determinants of universal health coverage. This situation is also related to the economic development of the countries [4]. In this context, poverty ratio, GDP growth and household expenditures play a significant role.

The concept of universal health coverage is especially important for developing economies for many different reasons. First of all, these countries are trying increase their economic and social conditions in order to become a developed country [5]. Within this context, universal health coverage allows these countries to achieve this objective by improving social factors. Additionally, because there is income inequality in these countries, people may have difficulties to access the necessary healthcare services. Moreover, since these countries usually have high populations, universal health coverage plays a significant role in these countries [6]. Finally, because of the lower economic power of the people in these countries, they may not be able to afford the necessary healthcare services.

It can be identified that performance measurement of universal health coverage is a crucial aspect. With the help of effective performance measurement, it can be much easier to understand the missing parts of these issues. Thus, qualified performance measurement methods should be taken into consideration. Owing to this condition, necessary recommendations can be presented to improve this process. In this circumstance, multicriteria decision-making models are usually preferred for performance measurement purposes since they consider many different significant factors at the same time [7,8,9]. Additionally, in this framework, the computational intelligence algorithms can be taken into the consideration to solve this problem [10,11,12,13,14,15]. Similar to this situation, an optimization algorithm can also be used for this purpose [16,17,18,19]. 

In this study, the aim is to measure the performance of universal health coverage in E7 economies. For this purpose, service- and financial-based criteria are defined by assessing the reports of World Health Organization and United Nations. Furthermore, with respect to the methodology, the fuzzy DEMATEL and MOORA methods are taken into the consideration to weigh these criteria and rank E7 countries regarding UHC.

It is thought that this study makes a contribution to the literature in many different ways. The elements of UHC and its analysis in emerging economies have been studied in the literature very little. In particular, the importance of the dimensions and criteria that affect UHC has not been studied in the literature beforehand. Similarly, there is a necessity to analyze the performance results and propose strategies in this context on a country-by-country basis. The main difference of this study is that universal health coverage of E7 economies is measured by considering both financial and non-financial items. In addition, considering this issue with fuzzy logic is another novelty of this study. 

In this study, there are basically five different sections. This section is the introductory part of the study and general information about the subject is shared in this section. In the second part, similar studies on the subject are examined and the missing areas in the literature are revealed. In the third part of the study, the methods used in the analysis process are given. The fourth part of the study includes the analysis. In the last section, the analysis results and recommendations are emphasized.

## 2. Literature Review

Universal health coverage is a very popular subject in the literature. Some studies have aimed to define the effects of health financing in the success of universal health coverage. For example, Dieleman et al. [5] considered the historical data of gross domestic product and health expenditures for 188 different countries. They established that health spending will increase to $20 trillion in 2040, so that there should be effective health financing. Similarly, Fahim et al. [6] also focused on the importance of health financing on universal health coverage in Bangladesh. They defined that with the better allocation of the funds, it can be possible to achieve a more effective health system. Additionally, Aso [1], Savedoff et al. [20], Borgonovi and Compagni [2], Agier et al. [21] and Alshamsan et al. [22] performed other studies that placed an emphasis on the significance of health financing in the success of the universal health coverage.

On the other side, the determinants of the universal health coverage are taken into the consideration by many different researchers. Rahman et al. [23] aimed to find the indicators of universal health coverage in Bangladesh. With the help of a Bayesian regression model, it is concluded that Bangladesh can achieve 80% of the target in 2030. Moreover, de Andrade et al. [4] also focused on the social determinants of universal health coverage for Latin American countries. They defined that political commitment plays a very crucial role in the performance of universal health coverage. In addition to them, Patel et al. [24] underlined the importance of technological factors in the performance of universal health coverage. This situation was also evaluated in some other studies in the literature [25,26,27].

Furthermore, the universal health coverage affects the health system was also analyzed by some researchers. Mboi et al. [28] tried to find the patterns of morbidity and mortality with the aim of understanding inequality in Indonesia. By focusing on GBD 2016 results, it is aimed to provide health coverage for all people who live in this country so that there can be effective health system. Additionally, Kruk et al. [29] focused on mortality caused by low-quality health systems in their study and reached the conclusion that it is possible to decrease mortality rates while increasing the quality in health systems by adopting universal health coverage programs. Also, Tangcharoensathien et al. [30] focused on the performance of the health system in Thailand. They reached a conclusion that in spite of the low GDP per capita, the health system performance in Thailand went up, especially after the implementation of universal health coverage. Citron et al. [31], Morgan et al. [32] and Kutzin [33] also focused on this issue in their studies.

In addition to them, the role of government or private institutions was assessed in some different studies. Miller et al. [34] made a study to define the effects of institutions on universal health coverage. For this context, data from 62 different countries for the years between 2000 and 2014 are taken into consideration. They reached a conclusion that inclusive institutional arrangements lead to more effective health systems and lower mortality rates. Lu and Chiang [35] analysed the ways of using health services supply effectively in order to provide universal health coverage in Taiwan. They reached the conclusion that public private partnerships in the health industry should be encouraged. In addition, the medical resource distribution should also be regulated. Awosusi et al. [36], Chemouni [3], Mcintyre et al. [37] and McPake and Hanson [38] also focused on this condition in their studies. 

This literature review shows that most of the studies focused on the economic aspects of UHC for different countries. However, there is no study in which universal health coverage is measured by considering both financial and non-financial issues at the same time. In addition to this issue, there is a need for a new study which provides weighted results for these factors because they can be guiding for academicians and state authorities. In this study, a new model is proposed to measure UHC by considering both financial and non-financial factors with the help of a different methodology, such as fuzzy logic. Hence, it is believed that this study can fill this gap in the literature.

## 3. Methods

### 3.1. Fuzzy Sets

The concept of fuzzy set is a class of objects with a continuum of grades of membership. Membership function is assigned to each object a grade of membership ranking between zero and one. This approach is introduced by Zadeh and applied for the various notions of inclusion, union, intersection, relation, and convexity [39]. Nowadays, it is widely used for complex decision making problems. Essential points of view for the fuzzy sets are provided as follows:

Let X be a space of objects with a generic element of X defined by x and X={x}. A fuzzy set A in X is a membership function $f_{A}\left(x\right)$ represents each point in X a real number in the interval [0, 1] with the value of $f_{A}\left(x\right)$ at x defines the grade of membership of x in A. In other words, the membership function and the fuzzy theory is based on this function. The numbers are identified as the subset with the confidence interval [40].

Nearer value of $f_{A}\left(x\right)$ and higher grade of membership of x in A are considered. When A is a set in the ordinary sense of the term, its membership function can take on only two values 0 and 1, with $f_{A}\left(x\right)=1\;\mathrm{or}\;0$ and reduces to the familiar characteristic function of a set A.

A fuzzy set is empty if and only if its membership function is identically zero on X. Two fuzzy sets A and B are equal, and defined as *A* = *B*, if and only if $f_{A}\left(x\right)=f_{B}\left(x\right)$ for all x in X. 

The complement of a fuzzy set A is defined by $\overset{´}{A}$ and formulated as:

(1) $$f_{\overset{´}{A}}=1-f_{A}$$

The notion of containment has a central role in the case of fuzzy sets. A is a subset of B, or A is smaller than or equal to B if and only if $f_{A}≦f_{B}$. In other words:

(2) $$A\subset B⟺f_{A}≦f_{B}$$

The union of two fuzzy sets A and B with the membership functions $f_{A}\left(x\right)$ and $f_{B}\left(x\right)$ is a fuzzy set C defined as follows:

(3) C=A∪B

and the membership function is:

(4) $$f_{C}\left(x\right)=Max\left[f_{A}\left(x\right),f_{B}\left(x\right)\right]$$

The intersection of two fuzzy sets A and B with the membership functions $f_{A}\left(x\right)$ and $f_{B}\left(x\right)$ is a fuzzy set C defined as follows:

(5) C=A∩B

and the membership function is:

(6) $$f_{C}\left(x\right)=Min\left[f_{A}\left(x\right),f_{B}\left(x\right)\right],\;x\in X$$

However, triangular fuzzy numbers are frequently applied in the multi-criteria making methods of real world problems. Some definitions are given below.

**Figure 1:**
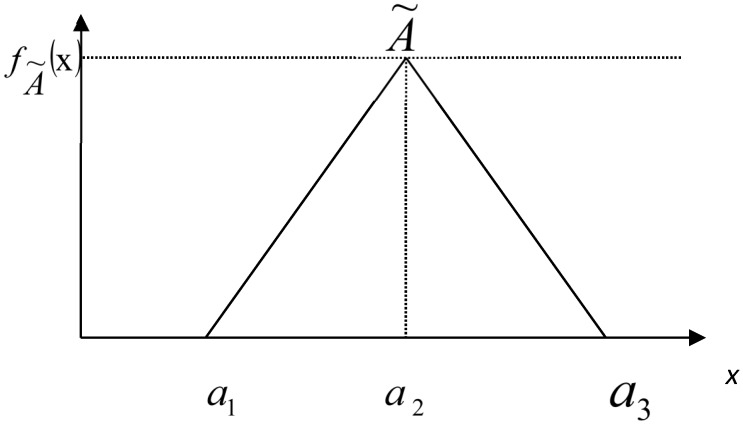
Membership function of the triangular fuzzy number.

Fuzzy numbers can generally be used as triangular fuzzy sets which can be represented as $\tilde{A}=\left(a_{1,}a_{2,}a_{3}\right)$. In this circumstance, *a*_1_ is smaller than *a*_2_ which is also lower than *a*_3_. Figure 1 gives information about the membership function of the triangular fuzzy sets.

In addition to them, Equation (7) explains the membership function $a_{1}$ of the fuzzy number $\tilde{A}$:

(7) $$f_{\tilde{A}}\left(X\right)=\{\begin{array}{cc} 0, & x<a_{1}\\ \left(x-a_{1}\right)/\left(a_{2}-a_{1}\right), & a_{1}\leq x\leq a_{2}\\ \left(a_{3}-x\right)/\left(a_{3}-a_{2}\right), & a_{2}\leq x\leq a_{3}\\ 0, & x>a_{3} \end{array}$$

### 3.2. DEMATEL

The expression of “decision making trial and evaluation laboratory” describes the acronym DEMATEL. Gabus and Fontela introduced this method in a research center in Genova and it is aimed at measuring the cause and effects factors of decision-making sets. Thus, the causality among the criteria could be defined more accurately [41,42]. Additionally, it is widely used for solving complex decision making problems [43,44]. There are several types of multicriteria decision-making approaches to measure the relative importance of factors. For example, Saaty [45] developed a analytic hierarchy process in terms of hierarchical conditions between the factors and the method is revised by considering the non-hierarchical relations defining the inner-dependency of the factors [46]. 

Additionally, the main benefit of the DEMATEL approach is that it can be possible to understand the impact relationship between the criteria. Moreover, these criteria can be weighted by using the DEMATEL method. Thus, it is possible to make two different analyses with this methodology. First of all, interdependence between the criteria can be identified [47,48,49]. There are mainly five stages in the calculation process. Firstly, linguistic evaluations are collected from the decision makers and converted into triangular fuzzy sets. After that, the initial direct relation fuzzy matrices of the decision makers are obtained and averaged values are considered to provide the direct relation matrix. In the following process, normalization procedure is applied to construct total fuzzy relation matrix. After the defuzzification process, the total row and column values of defuzzified total relation matrix are used for calculating the impact-relation degrees of each criterion as well as their relative weights.

DEMATEL methodology was considered for many different purposes in the literature. For instance, Abdel-Basset et al. [7], Kumar et al. [50] and Liu et al. [51] aimed to select the best supplier with the DEMATEL approach. On the other hand, Kaur et al. [52], Lin et al. [53], Li and Mathiyazhagan [54] and Luthra et al. [55] used this method to measure the performance of the supply chains. In addition to them, DEMATEL was also considered for assessing job satisfaction [56], exploring the indicators of environmentally oriented public procurement [57], identifying the barriers of remanufacturing [58], performance analysis [59,60,61], risk management [62] and evaluating the effectiveness of the knowledge transfer system [63]. 

### 3.3. MOORA

MOORA is another example of multi-criteria decision-making model. This approach was developed by Brauers and Zavadskas [64] with the aim of ranking different alternatives. The method is defined as the Multi-Objective Optimization on the basis of the Ratio Analysis [65] and used for the optimization of beneficial and non-beneficial criteria within definite limitations [66,67]. Similarly, TOPSIS and VIKOR are widely considered for ranking alternatives. TOPSIS was introduced by Hwang and Yoon [68] and used for determining the order of preference by similarity to the ideal solution and measuring the distances from the positive-ideal solution. VIKOR was firstly applied by Opricovic to define the compromise solutions in the ranking process of alternatives [69,70]. 

However, the main advantage of this method is that it takes a very short time to perform the necessary calculations and it is easy to implement. By considering this model, the criteria which have both positive and negative influences can be considered. In the computational procedures of MOORA, first of all, a decision matrix is constructed and a data set of alternatives is collected in terms of criteria. Then, a normalization procedure is applied to compute the positive and negative effects of the decision matrix. A weighted decision matrix is calculated by using the relative importance of each criterion. Finally, overall scores are determined to rank alternatives.

MOORA methodology is very popular in the literature. Thus, it was used by the researchers for different industries, such as logistics [71], manufacturing [72], finance [73,74,75], airlines [76] and health [8]. This method was also considered for supplier selection [9] and supply chain management [77,78].

## 4. Analysis 

**Figure 2:**
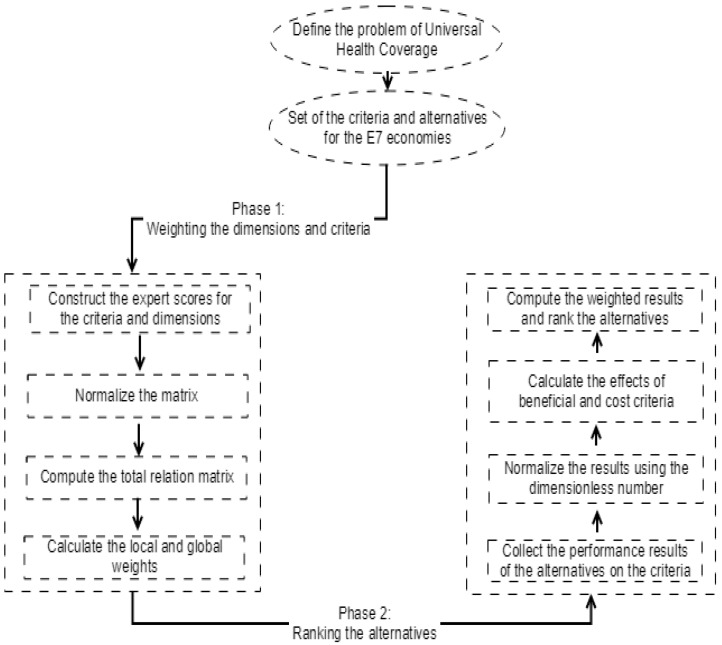
The flowchart of the hybrid decision making approach to the UHC.

In this study, universal health coverage performance is evaluated for E7 economies. In this context, there are two different phases in the analysis process. In the first phase selected dimensions and criteria are weighted by using the fuzzy DEMATEL approach. The impact relation degrees between different factors of universal health coverage are also illustrated. In the second stage, E7 countries are ranked to uncover their universal health coverage performance with the help of the MOORA method by using the selected data of countries. The model is applied using the formulas indicated in the methodology with the help of Microsoft Excel. The details of the proposed model are illustrated in Figure 2.

*Step 1*: Define the problem of multi-criteria decision-making approach. A set of dimension and criteria are defined for measuring the performance of universal health coverage in E7 economies. For this purpose, 14 different criteria are defined based on five different dimensions. In this process, the information regarding universal health coverage stated on the websites on World Health Organization and United Nations is taken into the consideration. The details are given on Table 1.

In the process of selection of these factors, service-based and financial based-items are considered. With respect to the service-based factors, three different dimensions are identified which are maternal and child health, non-communicable diseases and service capacity and access. The main reason for selecting these factors is that they are common issues in developing countries. On the other side, concerning financial-based items, catastrophic health spending and sustainable economic growth are analyzed. 

*Step 2*: Provide the linguistic evaluations for the dimensions and criteria. Three decision makers are appointed for obtaining the linguistic evaluations for each dimension and criterion. The decision makers are experts in the fields of medicine and health management with at least ten-years of experience. Five-point linguistic scales are used for evaluating the factors. Table 2 shows the linguistic scales and fuzzy numbers for measuring the dimensions and criteria.

The linguistic choices of decision makers for the dimensions and criteria are presented in Table 3, Table 4, Table 5, Table 6, Table 7, Table 8 and Table 9, respectively.

*Step 3*: Collect the dataset for the E7 economies. The data of these criteria in 2016 for E7 economies is listed in Table 10.

In Table 10, the averaged values of the E7 economies are considered for the data that is not available. For this purpose, the averaged values are computed for the criteria including at least two evaluation items. 

*Step 4*: Weigh the dimensions and criteria with the fuzzy DEMATEL method. In the first stage of the analysis, these dimensions and criteria are weighted using the fuzzy DEMATEL. The fuzzy DEMATEL calculation results are comprehensively provided in Appendix A. Overall analysis results are given in Table 11. 

Table 11 gives information indicating that both service-based and financial-based perspectives have equal weights. On the other side, regarding the service-based perspective, the dimension of service capacity and access (D3) has the highest importance. In addition to them, catastrophic out of pocket health spending (C9) is the most significant criterion. This situation explains that when people have health expenditures that are greater than 10% of their total income, it has a strong and negative influence on the universal health coverage performance. 

Moreover, it is also determined that pushed below an international poverty line (C10), annual growth rate of real GDP per capita (C12) and growth rates of household expenditure or income per capita (C14) are other important criteria which affect universal health coverage. It is obvious that macroeconomic conditions play a very significant role regarding this issue. In other words, in case of economic development of the countries, the performance of universal health coverage goes up.

**Figure 3:**
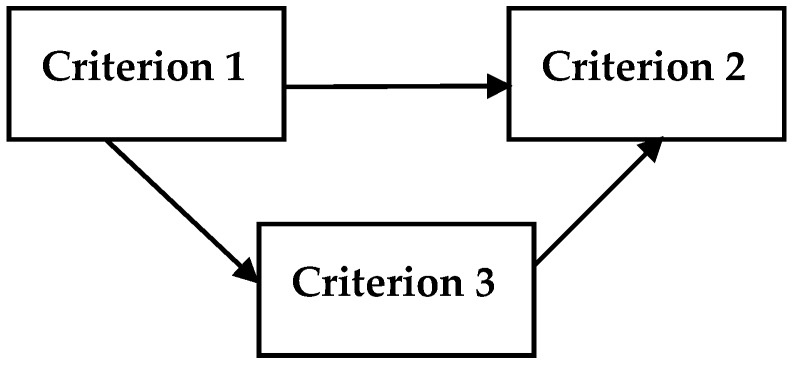
Impact-relation map for the criteria of maternal and child health.

**Figure 4:**
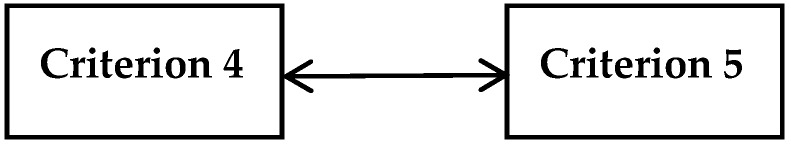
Impact-relation map for the criteria of noncommunicable diseases.

**Figure 5:**
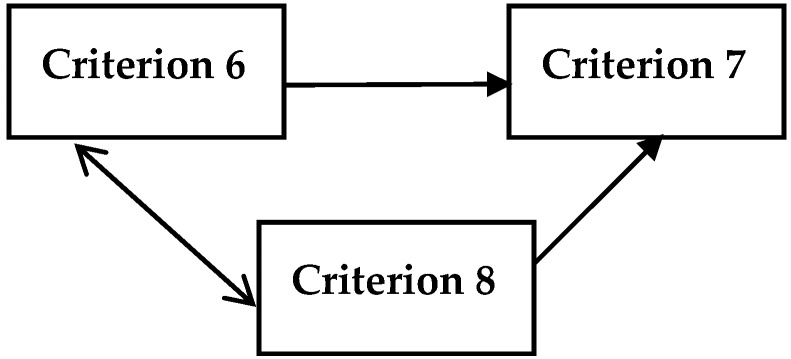
Impact-relation map for the criteria of service capacity and access.

Additionally, the impact and relation map for the criteria of each dimension is constructed with the fuzzy DEMATEL method to understand the degrees of influence and directions among the criteria of universal health coverage. The defuzzified values of the total relation matrix are used for computing the mutual relations between the criteria. For that, average value of matrix is determined as a threshold and the higher value than the threshold indicates that it has an influence on the other criterion. Accordingly, Figure 1, Figure 2, Figure 3, Figure 4 and Figure 5 illustrate the impact-relation maps for the dimensions of universal health coverage.

According to the impact and relation results of Figure 3, antenatal and delivery care (Criterion 1) has an impact on both full child immunization (Criterion 2) and health-seeking behaviour for child illness (Criterion 3) while Criterion 2 has no impact on the other factors. 

Figure 4 shows that there is a mutual relationship between prevalence of raised blood pressure (Criterion 4) and prevalence of raised blood glucose (Criterion 5).

In Figure 5, basic hospital access (Criterion 6) and access to essential medicines (Criterion 8) have a mutual impact between each other as health-worker density (Criterion 7) has no impact on the other criteria of service capacity and access. 

**Figure 6:**
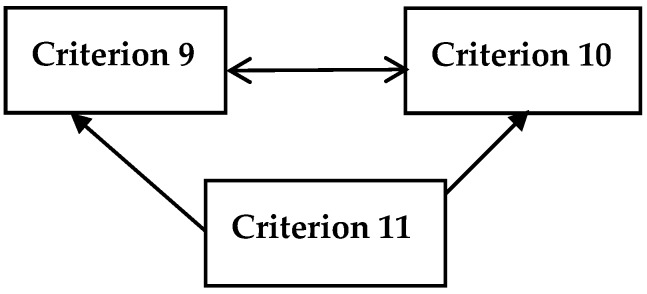
Impact-relation map for the criteria of catastrophic health spending.

Figure 6 represents that poverty gap due to out of pocket health spending (Criterion 11) influences both catastrophic out of pocket health spending (Criterion 9) and pushed below an international poverty line (Criterion 10) whereas Criteria 9 and 10 affect each other. 

**Figure 7:**
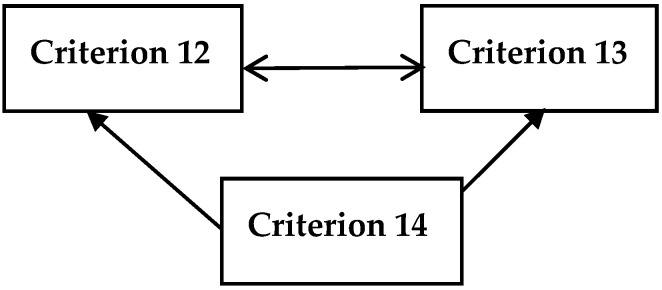
Impact-relation map for the criteria of sustainable economic growth.

Similarly, in Figure 7, growth rates of household expenditure or income per capita (Criterion 14) impacts annual growth rate of real GDP per capita (Criterion 12) and annual growth rate of real GDP per employed person (Criterion 13) systematically. However, Criterion 12 and 13 have a mutual effect among them. 

*Step 5*: Rank the alternatives with the MOORA method. Furthermore, in the second stage of the analysis, the performance of E7 economies is ranked with the help of MOORA approach. The MOORA computations are systematically presented in Appendix B. The ranking results are summarized in Table 12. 

Table 12 states that Russia is the country that has the highest universal health coverage performance. Moreover, Indonesia and Turkey are other countries which have highest performance as well. On the other hand, China, India and Brazil occupy the last ranks. It is thought that the countries with low GDP per capita have some problems with respect to the universal health coverage. In addition to them, it is also obvious that high population has also negative influence on the countries such as, China and India.

## 5. Conclusions

Universal health coverage refers to the situation where all citizens can access the necessary healthcare services for their lives. That is to say, it includes the people in the country who have lower income. Therefore, having an effective universal health coverage program is one of the most significant purposes of emerging economies due to the many different reasons, such as high income inequality, high population and the lower economic power of the people. It is obvious that the determinants of this system should be identified to increase the performance in these countries.

In this study, the aim was to measure universal health coverage in E7 economies. Within this context, five different dimensions and 14 different criteria are selected. In this process, the explanations of World Health Organization and United Nations regarding universal health coverage are taken into the consideration. While weighting the dimensions and criteria are considered with a fuzzy DEMATEL method and the, MOORA approach is used for ranking the universal health coverage performance of E7 economies.

According to the analysis results, it is defined that both service-based and financial-based perspectives have equal weights. In addition to this situation, it is also determined that the dimension of service capacity and access has the highest weight with respect to the service-based perspective. Another important conclusion is that catastrophic out of pocket health spending, being pushed below an international poverty line and annual growth rate of real GDP per capita are the most significant criteria for universal health coverage performance. Russia is the country that has the highest universal health coverage performance, whereas China, India and Brazil occupy the last ranks.

The findings give information that macroeconomic conditions play a very significant role on the performance of universal health coverage in E7 countries. In addition to them, it is also obvious that high population also has a negative influence on the countries such as China and India. This condition indicates that population planning is very necessary for these countries to improve universal health coverage. Hence, it is recommended that a program should be established to protect people against financial risks related to health expenditures by providing exemptions or lowering out-of-pocket expenditures. By considering both service- and financial-based factors and using an original methodology in this study, we aimed to make a contribution to the literature. 

The main limitation of this study is related to the scope of the analysis. In this study, only E7 countries are taken into consideration. On the other hand, in future studies, more developing countries can also be analyzed via different methodologies to provide beneficial results. In addition to them, because this subject is very important for all countries, an analysis can also be made for developed economies.

## Figures and Tables

**Table 1:** Service and financial-based factors of universal health coverage.

Dimensions	Criteria	Definition
**Service-Based**		
Maternal and Child Health (D1)	Antenatal and delivery Care (C1)	The percentage of women aged 15–49 with a live birth in a given time period that received antenatal care four or more times.
	Full child immunization (C2)	The percentage of one-year-olds who have received three doses of the combined diphtheria, tetanus toxoid and pertussis vaccine in a given year.
	Health-seeking behaviour for child illness (C3)	Percentage of children under 5 years of age with symptoms of pneumonia (cough and difficult breathing NOT due to a problem in the chest and a blocked nose) in the two weeks preceding the survey taken to an appropriate health facility or provider.
Noncommunicable Diseases (D2) (-)	Prevalence of raised blood pressure (C4)	Percent of defined population with raised blood pressure (systolic blood pressure ≥ 140 OR diastolic blood pressure ≥ 90).
	Prevalence of raised blood glucose (C5)	Percent of defined population with fasting glucose ≥126 mg/dl (7.0 mmol/L) or history of diagnosis with diabetes or use of insulin or oral hypoglycaemic drugs.
Service capacity and Access (D3)	Basic hospital Access (C6)	The number of hospital beds available per every 10 000 inhabitants in a population.
	Health-worker density (C7)	Number of medical doctors (physicians), including generalist and specialist medical practitioners, per 1 000 population.
	Access to essential medicines (C8)	Median percent availability of selected generic medicines in a sample of health facilities.
**Financial-Based**		
Catastrophic health spending (D4) (-)	Catastrophic out of pocket health spending (C9)	Percentage of population with household expenditures on health greater than 10% of total household expenditure or income
	Pushed below an international poverty line (C10)	Percentage of population pushed below the $1.90 a day poverty line by household health expenditures
	Poverty gap due to out of pocket health spending (C11)	Percentage of increase in poverty gap due to household health expenditures, expressed as a proportion of the $1.90 a day poverty line
Sustainable economic growth (D5)	Annual growth rate of real GDP per capita (C12)	Percentage of growth in real GDP per capita to measure the economic growth in accordance with national circumstances
	Annual growth rate of real GDP per employed person (C13)	Percentage of growth in real GDP per employed person to measure the level of economic productivity
	Growth rates of household expenditure or income per capita (C14)	Percentage of household expenditure or income per capita to understand the inequality and income growth among the countries

Source: World Health Organization and United Nations.

**Table 2:** Linguistic variables of the impact-relationship degrees.

Influence Level	Triangular Fuzzy Numbers		
No (N)	0	0	0.25
Low (L)	0	0.25	0.5
Medium (M)	0.25	0.5	0.75
High (H)	0.5	0.75	1
Very High (VH))	0.75	1	1

Source: Uygun et al. [79]; Uygun and Dede, [80]; Khorasaninejad et al. [81].

**Table 3:** Linguistic evaluations of decision makers for the service-based perspective.

	Maternal and Child Health (D1)			Noncommunicable Diseases (D2)			Service Capacity and Access (D3)		
	DM1	DM2	DM3	DM1	DM2	DM3	DM1	DM2	DM3
D1	-	-	-	L	M	M	M	H	H
D2	L	L	M	-	-	-	L	M	H
D3	M	M	H	M	M	H	-	-	-

**Table 4:** Linguistic evaluations of decision makers for the financial-based perspective.

	Catastrophic Health Spending (D4)			Sustainable Economic Growth (D5)		
	DM1	DM2	DM3	DM1	DM2	DM3
D4	-	-	-	M	M	H
D5	M	H	H	-	-	-

**Table 5:** Linguistic evaluations of decision makers for the dimension of maternal and child health.

	Antenatal and Delivery Care (C1)			Full Child Immunization (C2)			Health-seeking Behaviour for Child Illness (C3)		
	DM1	DM2	DM3	DM1	DM2	DM3	DM1	DM2	DM3
C1	-	-	-	M	M	H	M	M	M
C2	M	L	M	-	-	-	L	M	M
C3	L	M	M	M	M	M	-	-	-

**Table 6:** Linguistic evaluations of decision makers for the dimension of non-communicable diseases.

	Prevalence of Raised Blood Pressure (C4)			Prevalence of Raised Blood Glucose (C5)		
	DM1	DM2	DM3	DM1	DM2	DM3
C4	-	-	-	M	H	H
C5	M	M	H	-	-	-

**Table 7:** Linguistic evaluations of decision makers for the dimension of service capacity and access.

	Basic Hospital Access (C6)			Health-Worker Density (C7)			Access to Essential Medicines (C8)		
	DM1	DM2	DM3	DM1	DM2	DM3	DM1	DM2	DM3
C6	-	-	-	M	M	H	L	M	M
C7	M	M	L	-	-	-	L	L	M
C8	L	M	M	M	M	H	-	-	-

**Table 8:** Linguistic evaluations of decision makers for the dimension of catastrophic health spending.

	Catastrophic out of Pocket Health Spending (C9)			Pushed below an International Poverty Line (C10)			Poverty Gap due to out of Pocket Health Spending (C11)		
	DM1	DM2	DM3	DM1	DM2	DM3	DM1	DM2	DM3
C9	-	-	-	M	M	H	L	M	M
C10	H	M	VH	-	-	-	L	M	M
C11	H	M	H	M	M	M	-	-	-

**Table 9:** Linguistic evaluations of decision makers for the dimension of sustainable economic growth.

	Annual Growth Rate of Real GDP Per Capita (C12)			Annual Growth Rate of Real GDP Per Employed Person (C13)			Growth Rates of Household Expenditure or Income Per Capita (C14)		
	DM1	DM2	DM3	DM1	DM2	DM3	DM1	DM2	DM3
C12	-	-	-	M	M	M	L	M	M
C13	H	M	L	-	-	-	L	M	M
C14	H	M	H	M	M	H	-	-	-

**Table 10:** Dataset of universal health coverage for the E7 economies.

Criteria/Alternatives	A1 (Brazil)	A2 (China)	A3 (India)	A4 (Indonesia)	A5 (Mexico)	A6 (Russia)	A7 (Turkey)
Antenatal and delivery Care (C1)	90.9	-	51.2	83.5	94.3	78.3	88.9
Full child immunization (C2)	90.4	99	76.2	76.9	93	88.1	95.4
Health-seeking behaviour for child illness (C3)	49.7	-	73.2	75.3	73.1	-	37.3
Prevalance of raised blood pressure (C4)	23.3	19.2	25.8	23.8	19.7	27.2	20.3
Prevalance of raised blood glucose (C5)	8.3	8.8	8.7	7.7	11.2	7.7	13.6
Basic hospital Access (C6)	22	42	7	12	15	82	27
Health-worker density (C7)	1.852	1.812	0.758	0.201	2.231	3.975	1.749
Access to essential medicines (C8)	76.7	14.4	2.8	57.8	46.3	100	-
Catastrophic out of pocket health spending (C9)	25.56	17.71	17.33	3.61	7.13	4.87	3.1
Pushed below an international poverty line (C10)	1.04	2.13	4.16	0.07	0.28	0.01	0.09
Poverty gap due to out of pocket health spending (C11)	0.39	0.64	1.12	0.01	0.04	0.01	0.01
Annual growth rate of real GDP per capita (C12)	−4.4	6.8	5.9	3.8	1.6	−0.3	1.6
Annual growth rate of real GDP per employed person (C13)	1.6	5.2	1.2	4.2	0.4	12	4.3
Growth rates of household expenditure or income per capita (C14)	2.25	8.23	-	3.41	0.96	0.52	4.66

Source: World Health Organization and United Nations.

**Table 11:** Local and global weights for the dimensions and criteria of universal health coverage.

Perspectives	Local Weights	Dimensions	Local Weights	Criteria	Local Weights	Global Weights
Service-Based (P1)	0.5	D1	0.331	C1	0.337	0.056
				C2	0.334	0.055
				C3	0.328	0.054
		D2	0.312	C4	0.501	0.078
				C5	0.499	0.078
		D3	0.357	C6	0.335	0.059
				C7	0.339	0.060
				C8	0.326	0.058
Financial-Based (P2)	0.5	D4	0.499	C9	0.346	0.087
				C10	0.336	0.084
				C11	0.318	0.080
		D5	0.501	C12	0.335	0.084
				C13	0.328	0.083
				C14	0.337	0.085

**Table 12:** Benefit and cost values and ranking the E7 economies for universal health coverage.

Alternatives	Benefit Criteria	Cost Criteria	$Y_{i}^{}$	Ranking
A1 (Brazil)	0.109	0.158	−0.048	7
A2 (China)	0.195	0.170	0.025	5
A3 (India)	0.199	0.242	−0.043	6
A4 (Indonesia)	0.165	0.066	0.099	2
A5 (Mexico)	0.142	0.085	0.057	4
A6 (Russia)	0.248	0.073	0.176	1
A7 (Turkey)	0.156	0.079	0.077	3

## Abbreviations

E7	Emerging **7** countries

## Appendix A

**Table A1:** Averaged direct-relation fuzzy matrices.

	**D1**			**D2**			**D3**		
**Maternal and Child Health (D1)**	0.000	0.000	0.000	0.167	0.417	0.667	0.417	0.667	0.917
**Noncommunicable Diseases (D2)**	0.083	0.333	0.583	0.000	0.000	0.000	0.250	0.500	0.750
**Service capacity and Access (D3)**	0.333	0.583	0.833	0.333	0.583	0.833	0.000	0.000	0.000
	**D4**			**D5**					
**Catastrophic health spending (D4)**	0.000	0.000	0.000	0.333	0.583	0.833			
**Sustainable economic growth (D5)**	0.417	0.667	0.917	0.000	0.000	0.000			
	**C1**			**C2**			**C3**		
**Antenatal and delivery Care (C1)**	0.000	0.000	0.000	0.333	0.583	0.833	0.250	0.500	0.750
**Full child immunization (C2)**	0.167	0.417	0.667	0.000	0.000	0.000	0.167	0.417	0.667
**Health-seeking behaviour for child illness (C3)**	0.167	0.417	0.667	0.250	0.500	0.750	0.000	0.000	0.000
	**C4**			**C5**					
**Prevalence of raised blood pressure (C4)**	0.000	0.000	0.000	0.417	0.667	0.917			
**Prevalence of raised blood glucose (C5)**	0.333	0.583	0.833	0.000	0.000	0.000			
	**C6**			**C7**			**C8**		
**Basic hospital Access (C6)**	0.000	0.000	0.000	0.333	0.583	0.833	0.167	0.417	0.667
**Health-worker density (C7)**	0.167	0.417	0.667	0.000	0.000	0.000	0.083	0.333	0.583
**Access to essential medicines (C8)**	0.167	0.417	0.667	0.333	0.583	0.833	0.000	0.000	0.000
	**C9**			**C10**			**C11**		
**Catastrophic out of pocket health spending (C9)**	0.000	0.000	0.000	0.333	0.583	0.833	0.167	0.417	0.667
**Pushed below an international poverty line (C10)**	0.500	0.750	0.917	0.000	0.000	0.000	0.167	0.417	0.667
**Poverty gap out of pocket health spending (C11)**	0.417	0.667	0.917	0.250	0.500	0.750	0.000	0.000	0.000
	**C12**			**C13**			**C14**		
**Annual growth rate of real GDP per capita (C12)**	0.000	0.000	0.000	0.250	0.500	0.750	0.167	0.417	0.667
**Annual growth rate per employed person (C13)**	0.250	0.500	0.750	0.000	0.000	0.000	0.167	0.417	0.667
**Growth rates of household expenditure (C14)**	0.417	0.667	0.917	0.333	0.583	0.833	0.000	0.000	0.000

**Table A2:** Normalized Direct-Relation Fuzzy Matrices.

	**D1**			**D2**			**D3**		
**Maternal and Child Health (D1)**	0.000	0.000	0.000	0.100	0.250	0.400	0.250	0.400	0.550
**Noncommunicable Diseases (D2)**	0.050	0.200	0.350	0.000	0.000	0.000	0.150	0.300	0.450
**Service capacity and Access (D3)**	0.200	0.350	0.500	0.200	0.350	0.500	0.000	0.000	0.000
	**D4**			**D5**					
**Catastrophic health spending (D4)**	0.000	0.000	0.000	0.364	0.636	0.909			
**Sustainable economic growth (D5)**	0.455	0.727	1.000	0.000	0.000	0.000			
	**C1**			**C2**			**C3**		
**Antenatal and delivery Care (C1)**	0.000	0.000	0.000	0.211	0.368	0.526	0.158	0.316	0.474
**Full child immunization (C2)**	0.105	0.263	0.421	0.000	0.000	0.000	0.105	0.263	0.421
**Health-seeking behaviour for child illness (C3)**	0.105	0.263	0.421	0.158	0.316	0.474	0.000	0.000	0.000
	**C4**			**C5**					
**Prevalence of raised blood pressure (C4)**	0.000	0.000	0.000	0.455	0.727	1.000			
**Prevalence of raised blood glucose (C5)**	0.364	0.636	0.909	0.000	0.000	0.000			
	**C6**			**C7**			**C8**		
**Basic hospital Access (C6)**	0.000	0.000	0.000	0.222	0.389	0.556	0.111	0.278	0.444
**Health-worker density (C7)**	0.111	0.278	0.444	0.000	0.000	0.000	0.056	0.222	0.389
**Access to essential medicines (C8)**	0.111	0.278	0.444	0.222	0.389	0.556	0.000	0.000	0.000
	**C9**			**C10**			**C11**		
**Catastrophic out of pocket health spending (C9)**	0.000	0.000	0.000	0.200	0.350	0.500	0.100	0.250	0.400
**Pushed below an international poverty line (C10)**	0.300	0.450	0.550	0.000	0.000	0.000	0.100	0.250	0.400
**Poverty gap out of pocket health spending (C11)**	0.250	0.400	0.550	0.150	0.300	0.450	0.000	0.000	0.000
	**C12**			**C13**			**C14**		
**Annual growth rate of real GDP per capita (C12)**	0.000	0.000	0.000	0.143	0.286	0.429	0.095	0.238	0.381
**Annual growth rate per employed person (C13)**	0.143	0.286	0.429	0.000	0.000	0.000	0.095	0.238	0.381
**Growth rates of household expenditure (C14)**	0.238	0.381	0.524	0.190	0.333	0.476	0.000	0.000	0.000

**Table A3:** Total-relation fuzzy matrices.

	**D1**			**D2**			**D3**		
**Maternal and Child Health (D1)**	0.067	0.375	3.460	0.165	0.599	3.885	0.291	0.730	4.201
**Noncommunicable Diseases (D2)**	0.088	0.469	3.309	0.045	0.322	3.173	0.179	0.584	3.698
**Service capacity and Access (D3)**	0.231	0.645	3.885	0.242	0.672	4.029	0.094	0.460	3.950
	**D4**			**D5**					
**Catastrophic health spending (D4)**	0.198	0.862	10.00	0.436	1.185	10.00			
**Sustainable economic growth (D5)**	0.545	1.354	11.00	0.198	0.862	10.00			
	**C1**			**C2**			**C3**		
**Antenatal and delivery Care (C1)**	0.047	0.338	3.176	0.251	0.683	3.916	0.192	0.602	3.627
**Full child immunization (C2)**	0.124	0.485	3.121	0.047	0.338	3.176	0.130	0.505	3.237
**Health-seeking behaviour for child illness (C3)**	0.130	0.505	3.237	0.192	0.602	3.627	0.041	0.318	3.060
	**C4**			**C5**					
**Prevalence of raised blood pressure (C4)**	0.198	0.862	10.00	0.545	1.354	11.00			
**Prevalence of raised blood glucose (C5)**	0.436	1.185	10.00	0.198	0.862	10.00			
	**C6**			**C7**			**C8**		
**Basic hospital Access (C6)**	0.043	0.355	4.862	0.261	0.737	6.000	0.130	0.540	4.938
**Health-worker density (C7)**	0.124	0.503	4.615	0.043	0.368	5.000	0.072	0.444	4.385
**Access to essential medicines (C8)**	0.143	0.572	5.169	0.261	0.737	6.000	0.030	0.323	4.631
	**C9**			**C10**			**C11**		
**Catastrophic out of pocket health spending (C9)**	0.106	0.545	6.069	0.241	0.710	5.862	0.135	0.564	5.172
**Pushed below an international poverty line (C10)**	0.365	0.919	6.638	0.095	0.503	5.724	0.146	0.605	5.345
**Poverty gap out of pocket health spending (C11)**	0.331	0.894	6.875	0.225	0.735	6.250	0.056	0.407	5.250
	**C12**			**C13**			**C14**		
**Annual growth rate of real GDP per capita (C12)**	0.052	0.316	2.008	0.173	0.522	2.242	0.117	0.438	2.000
**Annual growth rate per employed person (C13)**	0.177	0.538	2.308	0.048	0.300	1.942	0.117	0.438	2.000
**Growth rates of household expenditure (C14)**	0.284	0.681	2.675	0.241	0.632	2.575	0.050	0.313	2.000

**Table A4:** Defuzzified total relation matrices and weights.

	**D1**	**D2**	**D3**	${\left({\tilde{D}}_{i}+{\tilde{R}}_{i}\right)}^{def}$	${\left({\tilde{D}}_{i}-{\tilde{R}}_{i}\right)}^{def}$	**Weights**
**Maternal and Child Health (D1)**	0.91	1.15	1.31	6.42	0.32	0.331
**Noncommunicable Diseases (D2)**	0.95	0.81	1.10	6.06	−0.33	0.312
**Service capacity and Access (D3)**	1.19	1.23	1.05	6.92	0.01	0.357
	**D4**	**D5**	${\left({\tilde{D}}_{i}+{\tilde{R}}_{i}\right)}^{def}$	${\left({\tilde{D}}_{i}-{\tilde{R}}_{i}\right)}^{def}$	**Weights**	
**Catastrophic health spending (D4)**	2.40	2.67	10.47	−0.31	0.499	
**Sustainable economic growth (D5)**	2.99	2.43	10.52	0.31	0.501	
	**C1**	**C2**	**C3**	${\left({\tilde{D}}_{i}+{\tilde{R}}_{i}\right)}^{def}$	${\left({\tilde{D}}_{i}-{\tilde{R}}_{i}\right)}^{def}$	**Weights**
**Antenatal and delivery Care (C1)**	0.83	1.22	1.11	5.89	0.44	0.337
**Full child immunization (C2)**	0.93	0.82	0.96	5.84	-0.44	0.334
**Health-seeking behaviour for child illness (C3)**	0.97	1.10	0.79	5.72	0.00	0.328
	**C4**	**C5**	${\left({\tilde{D}}_{i}+{\tilde{R}}_{i}\right)}^{def}$	${\left({\tilde{D}}_{i}-{\tilde{R}}_{i}\right)}^{def}$	**Weights**	
**Prevalence of raised blood pressure (C4)**	2.43	2.99	10.52	0.31	0.501	
**Prevalence of raised blood glucose (C5)**	2.67	2.40	10.47	−0.31	0.499	
	**C6**	**C7**	**C8**	${\left({\tilde{D}}_{i}+{\tilde{R}}_{i}\right)}^{def}$	${\left({\tilde{D}}_{i}-{\tilde{R}}_{i}\right)}^{def}$	**Weights**
**Basic hospital Access (C6)**	1.14	1.63	1.30	7.78	0.36	0.335
**Health-worker density (C7)**	1.21	1.15	1.12	7.88	−0.92	0.339
**Access to essential medicines (C8)**	1.37	1.63	1.07	7.57	0.57	0.326
	**C9**	**C10**	**C11**	${\left({\tilde{D}}_{i}+{\tilde{R}}_{i}\right)}^{def}$	${\left({\tilde{D}}_{i}-{\tilde{R}}_{i}\right)}^{def}$	**Weights**
**Catastrophic out of pocket health spending (C9)**	1.48	1.59	1.36	9.68	−0.84	0.346
**Pushed below an international poverty line (C10)**	1.88	1.40	1.43	9.39	0.04	0.336
**Poverty gap out of pocket health spending (C11)**	1.90	1.68	1.25	8.88	0.79	0.318
	**C12**	**C13**	**C14**	${\left({\tilde{D}}_{i}+{\tilde{R}}_{i}\right)}^{def}$	${\left({\tilde{D}}_{i}-{\tilde{R}}_{i}\right)}^{def}$	**Weights**
**Annual growth rate of real GDP per capita (C12)**	0.60	0.79	0.69	4.49	−0.32	0.335
**Annual growth rate per employed person (C13)**	0.82	0.58	0.69	4.40	−0.22	0.328
**Growth rates of household expenditure (C14)**	0.99	0.94	0.60	4.53	0.55	0.337

## Appendix B

**Table A5:** Dimensionless numbers for the alternatives.

Criteria/Alternatives	A1	A2	A3	A4	A5	A6	A7
**C1**	0.448	0.093	0.252	0.412	0.465	0.386	0.438
**C2**	0.385	0.421	0.324	0.327	0.396	0.375	0.406
**C3**	0.339	0.128	0.499	0.513	0.498	0.212	0.254
**C4**	0.384	0.316	0.425	0.392	0.325	0.448	0.334
**C5**	0.325	0.345	0.341	0.302	0.439	0.302	0.533
**C6**	0.219	0.418	0.070	0.119	0.149	0.815	0.268
**C7**	0.332	0.325	0.136	0.036	0.400	0.712	0.313
**C8**	0.516	0.097	0.019	0.389	0.311	0.673	0.154
**C9**	0.692	0.479	0.469	0.098	0.193	0.132	0.084
**C10**	0.217	0.444	0.867	0.015	0.058	0.002	0.019
**C11**	0.289	0.475	0.831	0.007	0.030	0.007	0.007
**C12**	−0.402	0.621	0.538	0.347	0.146	−0.027	0.146
**C13**	0.110	0.358	0.083	0.289	0.028	0.825	0.296
**C14**	0.095	0.349	0.898	0.145	0.041	0.022	0.198
